# Effects of dietary creatine supplementation on kidney and striated skeletal muscles of rats submitted to ischemia and reperfusion of hind limbs

**DOI:** 10.1590/ACB360305

**Published:** 2021-04-21

**Authors:** Antonio Augusto Moreira, Acácio Francisco, Fernanda Macedo dos Reis Moreira, Lawani Rigopoulos, Douglas Tsunemi, Marco Antônio Soufen

**Affiliations:** 1 PhD, Assistant Professor. Universidade de Mogi das Cruzes – College of Medicine – Department of Vascular Surgery – Mogi das Cruzes (SP), Brazil.; 2 Graduate student. Universidade de Mogi das Cruzes – College of Nutrition – Department of Vascular Surgery – Mogi das Cruzes (SP), Brazil.; 3 Graduate student. Universidade de Mogi das Cruzes – College of Medicine – Department of Vascular Surgery – Mogi das Cruzes (SP), Brazil.; 4 Graduate student. Universidade de Mogi das Cruzes – Department of Vascular Surgery – Mogi das Cruzes (SP), Brazil.; 5 PhD, Assistant Professor. Universidade de Mogi das Cruzes – College of Medicine – Department of Pathology – Mogi das Cruzes (SP), Brazil.

**Keywords:** Ischemia, Reperfusion, Creatine, Histology, Rats

## Abstract

**Purpose:**

To evaluate the effect of creatine supplementation in the diet of rats subjected to ischemia and reperfusion of hind limbs.

**Methods:**

Eighteen male Wistar rats were randomized to receive dietary creatine supplementation (G1) or no supplementation (G2), before being subjected to 4 h of ischemia followed by 4 h of reperfusion. In addition, 10 rats (G3) underwent the same surgical procedure, without ischemia, but with supplementation. After reperfusion, kidney and musculature were evaluated for histological damage and serum levels of alanine aminotransferase, urea and creatinine were obtained.

**Results:**

The urea dosage showed significant differences between the groups (averages G1 = 155.1; G2 = 211.27; G3 = 160.42). Histological analysis found significant differences between G1 and G2 (but not between G1 and G3) in renal myoglobin cylinders and vacuolar degeneration variables and in hypereosinophilia and karyopyknosis variables in muscle fibers. There were no significant differences in the other variables studied.

**Conclusions:**

Creatine supplementation was related to fewer histological lesions, as well as lower levels of plasma urea, which may suggest a protective effect against lesions caused by ischemia and reperfusion of posterior paws muscles in Wistar rats.

## Introduction

Myopathic-nephrotic-metabolic syndrome or reperfusion syndrome contributes significantly to the increase in morbidity and mortality from ischemic injuries of several organs^1^^–^ 3. These injuries result from oxidative stress and the inflammatory response, which appear after intervals of only 30 min, and irreversible changes in skeletal muscle occur after 4 to 6 h^4^^,^ 5.

Renal ischemia during arterial occlusion, shock, organ transplantation and arterial clamping for the treatment of nonocclusive arterial diseases, such as aneurysms, are commonly associated with cell death and early reperfusion remains a first-line strategy to minimize damage^6^. It is essential to investigate strategies that can be used at the time of reperfusion to prevent this type of injury, including creatine^7^^,^ 8. When creatine is consumed orally, it is absorbed intact by the intestinal epithelium and unchanged by gastric acid secretion. Its supplementation with a high dose (300 mg/kg/day of body weight) for a period of five to seven days leads to a rapid increase of intramuscular creatine, improving the working capacity of skeletal muscles and delaying the onset of muscle fatigue^9^.

The energy supplied through the creatine/adenosine triphosphate/creatine kinase (Cr/ATP/CK) system for the protection of myocytes showed an increase in this energy supply, both in the damage caused in the ischemia and reperfusion phase, which can be an objective in protecting against the injuries caused by ischemia and after revascularization^10^.

However, there are still many doubts regarding this issue, which makes the subject widely discussed in the last 20 years^9^^,^ 11. The aim of this study was to evaluate the effects of dietary creatine supplementation on kidney and striated skeletal musculature of rats submitted to ischemia and reperfusion of hind limbs.

## Methods

This experimental trial was carried out between August and September 2019. This research proposal was approved by the Ethics Committee on the Use of Animals CEUA of the Universidade de Mogi das Cruzes in a meeting on the date of November 28, 2018 under number 013/2018 to include 36 adult male Wistar rats aged 10 months and average body weight of approximately 300 g each, supplied by the Central Animal Hospital of the Universidade de Mogi das Cruzes.

Twenty-nine animals aging 10 months were randomly divided by batch into three groups: experiment group (group 1), nine animals, which received creatine monohydrate supplementation (Creatine Monohydrate Micronized – Atlhetica Nutrition, Matão/SP) in the dose 2 g diluted in 500 mL of water 5 days before being subjected to a period of 4 h of ischemia and 4 h of reperfusion; control group (group 2), 10 animals that did not receive creatine supplementation with the same period of ischemia–reperfusion; and sham group (group 3), 10 animals that received supplementation but were not subjected to ischemia–reperfusion in order to verify if creatine supplementation could cause changes in the studied variables^12^^,^ 13.

### Experimental sequence

Each animal was weighed and anesthetized with zolazepam/tiletamine (Zoletil – Virbac, São Paulo/SP) at a dose of 20 mg/kg intramuscularly in the right quadriceps.

A median laparotomy of 4 cm in length was performed and the aorta was connected immediately below the emergence of the left renal artery with 7.0 propylene suture in animals in groups 1 and 2. The 7.0 propylene sutures were passed around the group 3 animals’ infrarenal aorta, but no ligation was performed to interrupt aortic flow in animals in this group^14^.

The efficacy of the ligation was detected by the appearance of pallor, cyanosis, decreased temperature in the hind legs, by the absence of pulse and flow from the aorta below the ligation, confirmed by intraoperative doppler flowmetry.

After closing the abdominal wall with 3.0 cotton thread, postoperative analgesia was performed with butorphanol at a dose of 20 mg/kg subcutaneously in the nuchal region.

After the 4-hour period of ischemia, the animals were again anesthetized and submitted to removal of the aortic ligation in groups 1 and 2, as well as the 7.0 propylene thread around the aorta in group 3.

The abdominal wall was closed again to comply with the 4-hour reperfusion period.

After this period of reperfusion, the left nephrectomy was performed, the left posterior paw muscle was removed and cardiac puncture was performed to collect 5 mL of blood, a determining factor of euthanasia^15^. The blood was transferred to a dry test tube, which was numbered and sent to the laboratory for processing the serum dosages of urea, creatinine and alanine aminotransferase (ALT). The allocation secret was obeyed.

### Sample preparation

Kidney and muscle block were fixed in 10% buffered formalin solution for inclusion in paraffin and underwent5 μm cuts and stained using the hematoxylin-eosin technique.

### Histological analysis

**Figure 1:**
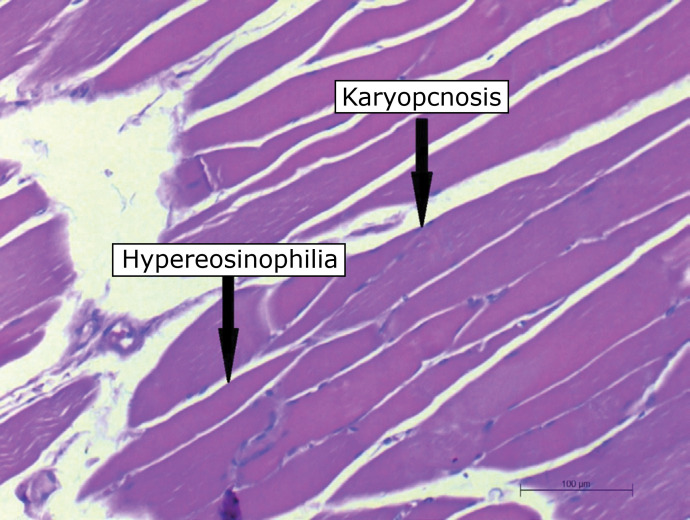
Histological slide demonstrating alterations assessed in the musculature of the posterior limbs (image is increased by 200 ×).

**Figure 2:**
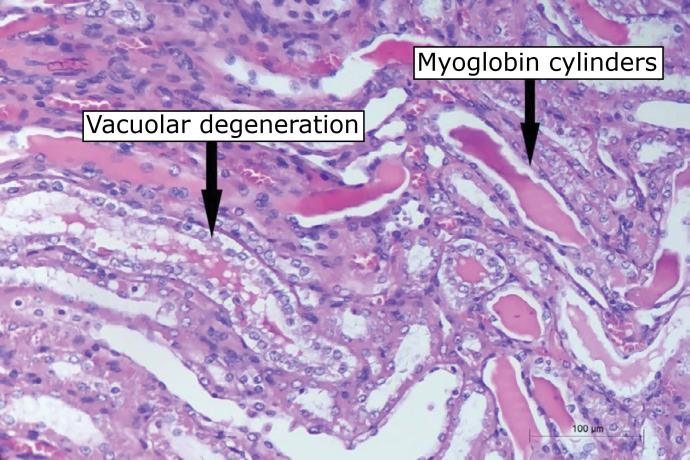
Histological slide demonstrating kidney injury markers assessed in the present study (image is increased by 200 ×).

The blocks were numbered and sent for histological analysis of the interstitial edema variables, hypereosinophilia of muscle fibers, inflammatory infiltrate, karyopyknosis (Fig. 1) and necrosis for the muscles of the posterior members, and the presence of myoglobin cylinders, vacuolar degeneration of tubular cells and acute tubular necrosis for the kidney (Fig. 2) by a pathologist in a blind test.

### Histological quantification

To quantify the histological variables, 10 microscopic fields were analyzed in a 400-fold increase that did not match in each sample. The results of all variables were expressed in semiquantitative form in percentages, according tothe ratio of expression of the changes in relation to the total observed, as follows: 0 = absent; 1 = less than 10%;2 = 11 to 25%; 3 = 26 to 50%; and 4 = more than 51%.

### Statistical analysis

The results were tabulated and submitted to the Shapiro–Wilk normality test, and when the normal distribution of the sample was proven, the analysis of variance (ANOVA) test was used and, otherwise, the Kruskal–Wallis nonparametric analysis of variance test was used. P ≤ 0.05 was defined for significance.

## Results

The average weight of the Wistar rats was 445.7516 g (± 58.6). The ANOVA test did not show significant differences between groups.

The ANOVA test demonstrated a significant increase in plasma urea levels (p < 0.05) in the group that did not receive dietary creatine supplementation when compared to the experiment group (Table 1). There was no significant difference when the values of group 1 with group 3 were compared (p > 0.05). Despite the higher value presented by the control group, there was no significant difference between the plasma creatinine values (p > 0.05) when comparing the three groups (Table 1). There was also no significant difference (p > 0.05) in the values found between the three groups studied, with the highest values being shown in group 2 (Table 1).

**Table 1:** Plasma dosage averages and standard deviations.

	Urea	Creatine	ALT
Group 1	155.10 (47.06)	1.27 (0.00)	21.66 (9.60)
Group 2	211.27 (40.39)	1.46 (0.32)	32.01 (13.99)
Group 3	160.42 (39.69)	1.37 (0.00)	20.85 (13.39)
ANOVA	p = 0.012	p > 0.05	p > 0.05

The Kruskal–Wallis test demonstrated a significant increase (p < 0.05) in the amount of myoglobin cylinders in the kidneys of rats in the group that did not receive dietary creatine supplementation when compared to the experiment group (Table 2). There was no significant difference when the values of group 1 were compared with group 3 (p > 0.05).

**Table 2:** Averages and standard deviations of renal and muscle anatomopathological variables.

Group	Myoglobin cylinders (kidney)	Vacuolar degeneration (kidney)	Hypereosinophilia of muscle fibers	Karyopyknosis in muscle fibers
1	0.78 (0.83)	0.33 (0.50)	0.11 (0.33)	0.11 (0.33)
2	2.11 (1.05)	1.22 (0.44)	1.00 (0.00)	0.88 (0.33)
3	0.70 (1.06)	0.40 (0.51)	0.30 (0.48)	0.30 (0.48)
Kruskal–Wallis	p = 0.02	p = 0.01	p = 0.01	p = 0.01

There was a higher incidence of vacuolar degeneration in the kidney of rats that did not receive dietary supplementation (p < 0.05) when compared to the other groups (Table 2).

The renal anatomopathological variable tubular necrosis did not show significant changes in any sample, in any group studied.

The group that did not receive dietary creatine supplementation showed more muscle fiber hypereosinophilia (Table 2) when compared to the other groups (p < 0.05).

Table 2 shows a greater presence of karyopyknosis in the posterior leg muscle cells of rats that did not receive dietary creatine supplementation in relation to the other groups (p < 0.05).

The anatomopathological muscle variables, interstitial edema, inflammatory infiltrate and necrosis did not show significant changes in any of the samples of any group studied.

## Discussion

Oral creatine supplementation is related to an increase in the working capacity of skeletal muscles, delaying the onset of muscle fatigue^9^. This is due to the energy supplied through the Cr/ATP/CK system, which can promote protection against cell damage caused by ischemia in various organs, such as the kidneys, lungs, heart, brain, intestine and testicles^3^^,^ 6 ^,^ 10 ^,^ 16 ^,^ 17 ^,^ 18.

The lack of randomized controlled studies using creatine supplementation to assess muscle and kidney injuries after ischemia and reperfusion has motivated the purpose of this research. It was hypothesized that creatine supplementation could reduce injuries caused by acute muscle ischemia and consequently kidney damage due to reperfusion.

There are some studies, such as the done by Pritchard and Kalra’s^11^, where creatine supplementation is related to decreased renal function, which led to the inclusion of the sham group in this study.

The analysis of renal function through the measurement of plasma urea and creatinine is frequently used in experimental models of ischemia and reperfusion^15^^,^ 19. The significant increase in plasma urea concentration (Table 1) in the group, which did not receive creatine supplementation (G2), may be related to a protective effect against renal reperfusion injuries. The nonsignificant difference in urea concentration between the experiment group (G1) and the sham group (G3) demonstrates that creatine supplementation did not cause significant kidney damage in the model studied.

There was a difference between the plasmatic concentration of creatinine and ALT between G1 and G2, but not between G1 and G3; however, the statistical analysis did not prove to be significant. This may be due to the smaller numerical variation of the studied values, which could perhaps be corrected by increasing the sample size, as well as a study that showed perioperative acute renal failure in patients undergoing myocardial revascularization after analysis of 2672 patients^20^.

A recent study shows a similar result with L-arginine supplementation, showing a significant protective effect on the kidneys of rats in terms of renal dysfunction, renal pathological changes, oxidative stress and nitric oxide imbalance. The same study indicates that L-arginine offers potential therapy in the prevention and treatment of ischemic kidney damage^21^.

In agreement with the biochemical analysis, the findings of the histological study demonstrated significant differences in renal myoglobin cylinders and vacuolar degeneration variables between G1 and G2, but not between G1 and G3. The renal variable tubular necrosis did not show significant differences between the groups, perhaps because it is a change, which occurs later in relation to the others^19^.

Muscle histological analysis also showed agreement with renal biochemical and histological results, showing significant differences between G1 and G2 and not between G1 and G3 in the variables hypereosinophilia and muscle fiber karyopyknosis, corroborating for a possible protective effect of creatine supplementation against ischemic injuries in muscle fibers. There were no significant differences between groups in muscle variables (interstitial edema, inflammatory infiltrate and necrosis), perhaps because these changes are more closely related to the time of ischemia and reperfusion^14^.

The reduction in muscle damage after creatine supplementation highlighted in the present study agree with the results of the experiment carried out by Cooke et al., which also aimed to evaluate its myoprotective effect, after chemically induced damage, concluding that the muscles supplemented with creatine had a higher proportion of undamaged fibers, as well as larger areas of regenerating fibers^13^.

## Conclusions

Dietary creatine supplementation was related to less muscle and kidney damage in histological analysis, as well as a lower amount of plasma urea in the biochemical study. These data may suggest a protective effect against injuries caused by ischemia and reperfusion of the hind leg muscles in Wistar rats.
